# Cases report: Mosaic structural variants of the *EXT1* gene in previously genetically unconfirmed multiple osteochondromas

**DOI:** 10.3389/fgene.2024.1435493

**Published:** 2024-08-13

**Authors:** Artem Borovikov, Andrey Marakhonov, Aysylu Murtazina, Kseniya Davydenko, Alexandra Filatova, Nailya Galeeva, Varvara Kadnikova, Natalya Ogorodova, Daria Gorodilova, Ilya Kanivets, Denis Pyankov, Konstantin Zherdev, Aleksandr Petel’guzov, Pavel Zubkov, Alexander Polyakov, Olga Shchagina, Mikhail Skoblov

**Affiliations:** ^1^ Research Centre for Medical Genetics, Moscow, Russia; ^2^ Genomed, Moscow, Russia; ^3^ Federal State Budgetary Educational Institution, Further Professional Education, Russian Medical Academy of Continuous Professional Education, Ministry of Healthcare of the Russian Federation, Moscow, Russia; ^4^ National Medical Research Center of Children’s Health, Moscow, Russia; ^5^ Department of Pediatric Surgery and Urology-Andrology, I. M. Sechenov First Moscow State Medical University, Moscow, Russia

**Keywords:** multiple osteochondromas, exostosis, *EXT1*, *EXT2*, mosaic deletion, genome sequencing

## Abstract

Multiple osteochondromas (MO) is a rare autosomal dominant skeletal disorder characterized by the development of multiple benign tumors known as osteochondromas. The condition is predominantly caused by loss-of-function variants in the *EXT1* or *EXT2* genes, facilitating relatively precise clinical diagnosis through established diagnostic criteria. Despite this, a notable percentage of MO cases (10%–20%) remains unresolved after sequencing coding regions and copy number analysis of both genes. In our study, we identified mosaic structural variants in two patients who initially yielded negative results on standard genetic analysis for MO. Specifically, mosaic deletions affecting exons 8–11 and exons 2–11 in the *EXT1* gene were detected. RNA analysis was performed in one case, while both cases underwent genome sequencing. To date, only six mosaic copy number variations have been reported in association with MO, representing a minority among known variants in both genes. Our report provides a detailed analysis of these findings, highlighting the significance of advanced genetic testing techniques in detecting mosaic variants in the *EXT1/2* genes.

## 1 Introduction

Multiple osteochondromas (MO), ICD-11: LD24.20, is a rare autosomal dominant skeletal disorder characterized by the formation of multiple benign tumors known as osteochondromas (Bovée, 2008; International Classification of Diseases, Eleventh Revision, 2019/2021). The disease is primarily caused by heterozygous loss-of-function variants in the *EXT1* or *EXT2* genes (Ahn et al., 1995; Stickens et al., 1996). Around 10%–20% of MO cases remain undiagnosed even after investigating the single-nucleotide variants (SNVs) within the coding regions and conducting copy number variation (CNV) analysis in the *EXT1* and *EXT2* genes (Ciavarella et al., 2013; Santos et al., 2018; Caino et al., 2022; Güneş et al., 2023). In our previous recent investigation involving a cohort of 244 families initially diagnosed with MO, we observed that 3.6% of these cases could be attributed to loss-of-function mutations in the *PTPN11* gene (Borovikov et al., 2024). However, this finding requires confirmation by further cohort studies.

Other potential causes of undiagnosed cases could include deep intronic variants or variants in regulatory regions of the *EXT1/2* genes, which are not routinely analyzed, and no such variants have been described to date (Stenson et al., 2003). Additionally, mosaic variants may contribute to disease pathology, as evidenced by six reported cases of mosaic CNVs (Szuhai et al., 2010; Sarrión et al., 2013; Oliver et al., 2019).

We identified mosaic structural variants in two patients whose routine genetic tests yielded negative findings, utilizing RNA analysis in one case and genome sequencing in both cases. In this report, we provide a detailed description of these two cases.

## 2 Materials and methods

### 2.1 Clinical data

The diagnosis of MO was based on typical clinical and/or radiographic findings (Bovée, 2008). Clinical examinations and scale classification perform according to the scale of severity of classification proposed by Istituto Ortopedico Rizzoli (IOR) (Mordenti et al., 2021).

### 2.2 DNA analysis

DNA was extracted from whole-blood samples using a Wizard^®^ Genomic DNA Purification Kit (Promega, United States). Sanger sequencing was carried out using the ABI PRISM 3500 Genetic Analyzer (Applied Biosystems, Foster City, CA, United States). A custom NGS panel, consisting of three genes, NM_000127.3 (*EXT1*), NM_207122.2 (*EXT2*), and NM_002834.5 (*PTPN11*), was used for sequencing on an Ion S5 (Thermo Fisher Scientific, Waltham, MA, United States) or MiSeq (Illumina, United States) sequencer. CNV analysis was carried out using the SALSA MLPA Probemix P215-B4 EXT (MRC Holland). Genome sequencing (GS) was performed using a DNBSEQ-G400 instrument in a pair-ended mode (2 × 150 bp) with an average on-target coverage of 30× with a DNBSEQ™ True PCR-Free kit (BGI, Beijing, China) for library preparation (GenoMed Ltd., Moscow, Russia). WGS findings were confirmed by Sanger sequencing.

### 2.3 Bioinformatics analysis

Bioinformatics analysis was performed using an in-house software pipeline described earlier with modifications (Rudenskaya et al., 2019). In brief, it included quality control of raw reads (FastQC tool v0.11.5), followed by read mapping to the hg19 human genome assembly (minimap2 v2.24-r1122), sorting of the alignments, and marking duplicates (Picard Toolkit v2.18.14). Base recalibration and variant calling were performed with GATK3.8. Variant annotation was done using ANNOVAR (v2018Apr16). CNV and SV analysis was performed using the Manta tool (v1.6.0).

### 2.4 RNA analysis

mRNA analysis was performed on primary cultured fibroblasts (PMBCs) isolated from the patient. Total RNA was extracted using the ExtractRNA reagent (Evrogen, Russia) according to the manufacturer’s recommendations. Reverse transcription was carried out using the ImProm-II™ Reverse Transcription System (Promega, United States). *EXT1* and *EXT2* mRNAs were divided into six overlapping loci each. The mRNA exonic structure was analyzed by PCR with locus-specific primers. Isoforms of EXT1/EXT2 were compared with mRNA isoforms from healthy donors. The PCR products were analyzed by gel electrophoresis, followed by Sanger sequencing. All loci were examined additionally by NGS. Libraries were prepared and sequenced on an Ion Torrent S5 system (with coverage >200,000). The raw sequencing data were processed with a custom pipeline based on open-source bioinformatics tools HISAT2, SAMtools, and SAJR. Splice junctions were visualized using a Sashimi plot in the IGV browser.

## 3 Case reports

### 3.1 Case 1

**FIGURE 1:**
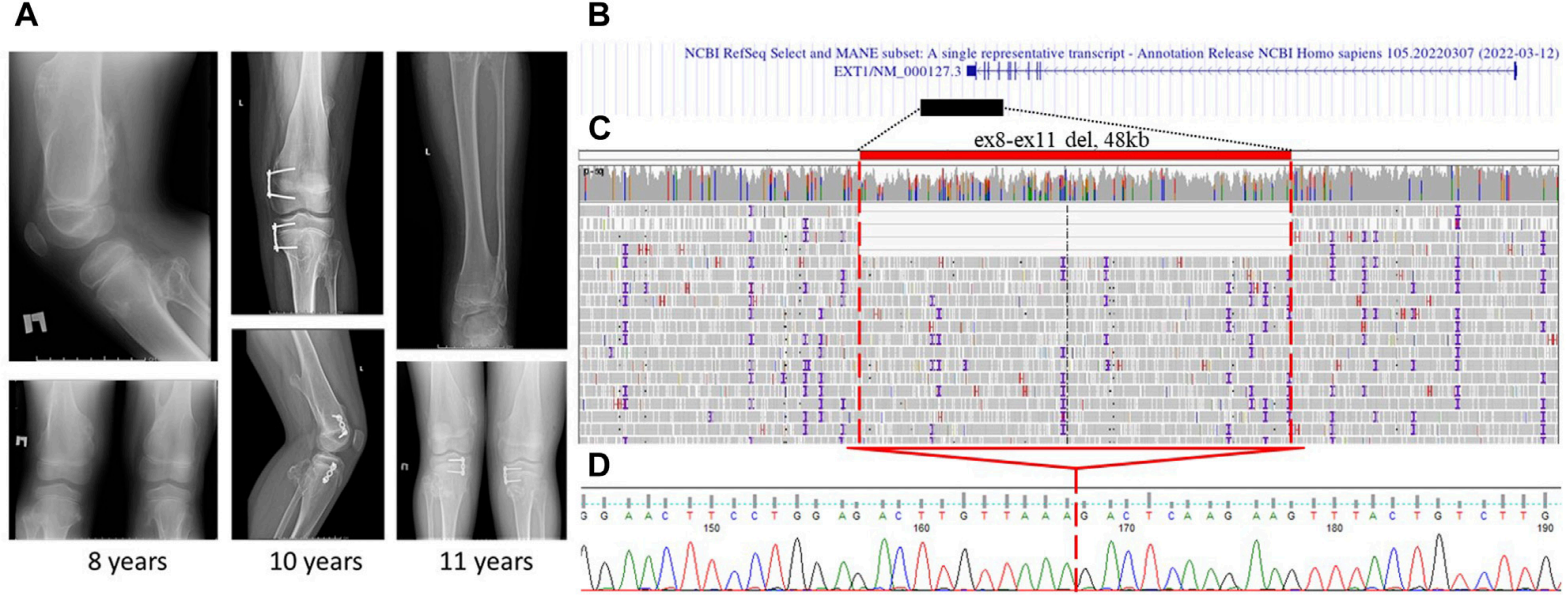
Case 1: **(A)** X-ray images of proband 1 at the age of 8, 10, and 11 years. Multiple osteochondromas around the knee and on the femur are shown. **(B)** Alignment of the deletion of exons 8–11 in the *EXT1* gene in the UCSC browser was found. **(C)** GS data on mosaic gross deletion from case 1 visualized in the IGV browser. **(D)** Chromatogram showing the region encompassing the breakpoints of the deletion, with a red line marking the borders of the deletion.

The first proband was an 11-year-old male child who exhibited dense bony formations of varying severities on the chest and extremity bones. The initial protrusion was discovered at the age of 3 years in the distal part of the popliteal fossa. Subsequently, the patient’s relatives observed the appearance of similar formations in the shoulders, hips, ribs, and forearm. Radiography of the knee, hip joints, and upper extremities at the age of 8–11 years revealed multiple osteochondromas in the popliteal fossa, distal part of the ulna, and around the hip joint (Figure 1A). The proband underwent several surgeries.

Clinical examination showed a normal facial appearance and psychomotor development. However, the patient presented with hallux valgus deformity of the feet, weight-bearing imbalance when resting on the left leg, and tenderness during active movements of the left knee and hip joints. Palpation revealed dense formations on the posterior surface of the proximal part of the left lower leg, around the left scapula, wrist, and ribs. Additionally, there was ulnar deviation of both hands due to deformities in both radial bones. According to the IOR score, his clinical severity was categorized as IIB.

Targeted sequencing of the exon-coding regions by direct Sanger sequencing and MLPA of the *EXT1* and *EXT2* loci yielded negative results. The next step involved conducting RT-PCR on *EXT1/2* genes to analyze the exon–exon structure and gene expression. This revealed a borderline unequal expression of alleles (45% for the reference allele G and 55% for the alternative allele A) at the common SNP (rs17439693) in exon 6 of the *EXT1* gene and the absence of splicing aberrations in both genes. Notably, this SNP was the only heterozygous variant in the coding regions of the *EXT1/2* genes. Following negative RNA analysis results, direct sequencing of the 5′untranslated and promoter region also did not reveal any variants. Subsequent GS was performed, which identified a mosaic deletion of chr8:118780297-118828217del (4/40 = 10.0% of the total read count) involving exons 8–11 of the *EXT1* gene (Figures 1B, C). This finding was further confirmed by Sanger sequencing (Figure 1D).

### 3.2 Case 2

**FIGURE 2:**
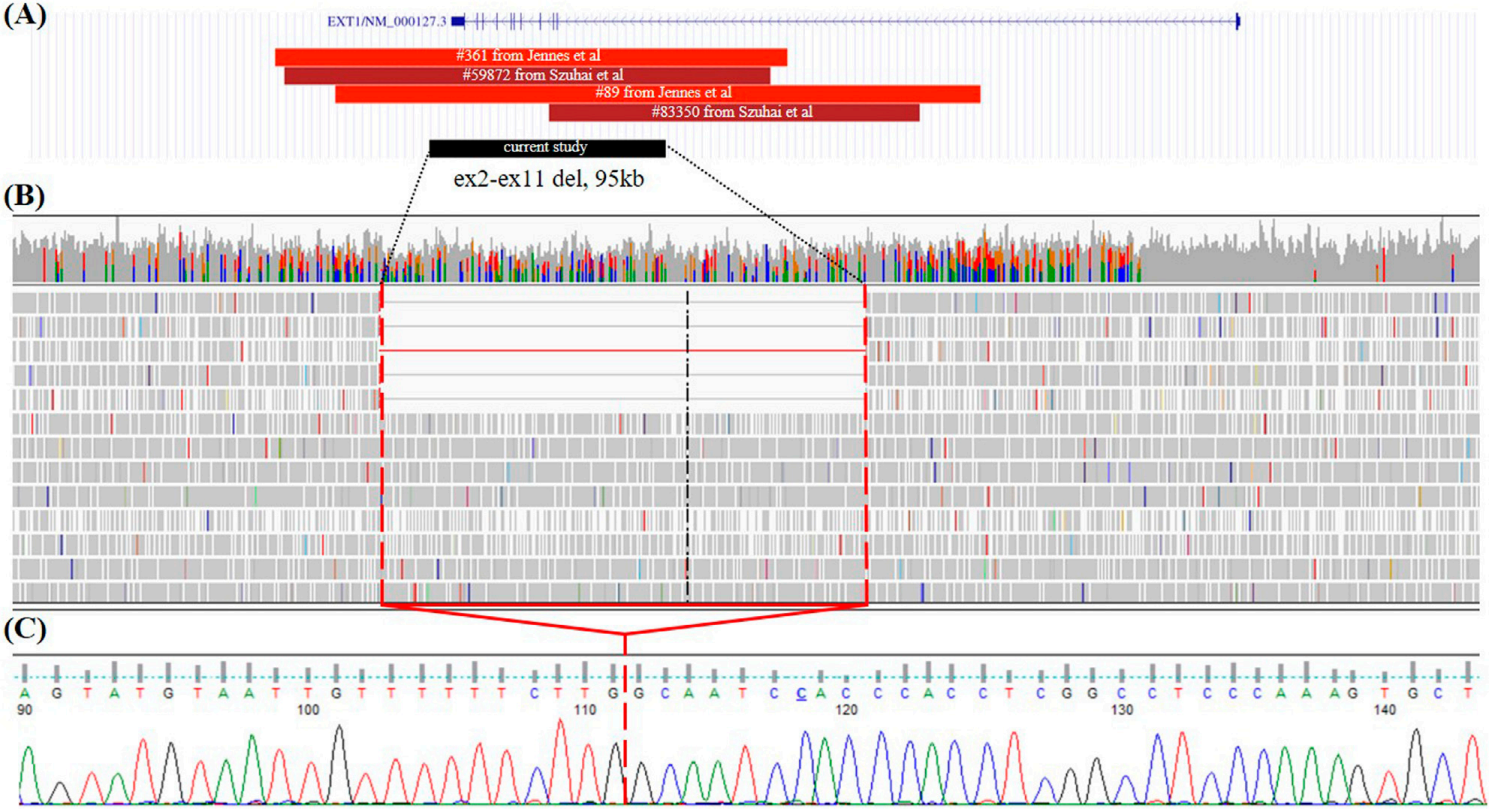
Case 2. **(A)** Alignment of all deletion of exons 2–11 in the *EXT1* gene with known breakpoint borders on the UCSC browser from the literature and case 2 from the current study. Red—germline CNVs; dark red—mosaic CNVs; black—deletion found in the present study. **(B)** GS data on mosaic gross deletion from case 2 visualized in the IGV browser. **(C)** Chromatogram showing the region encompassing the breakpoints of the deletion, with a red line marking the borders of the deletion.

In another proband, the first osteochondroma was detected at 3.5 years in the left forearm area. Over 1.5 years of observation, additional small osteochondromas appeared in other parts of the body. Surgical interventions were not performed; however, at the time of examination, surgical removal of the osteochondroma in the forearm area was planned. A clinical examination conducted at the age of 5 years revealed dense formations in the distal region of the left forearm, in the area of the left knee joint, and small formations in the area of the right humerus. The OIR score was IA, which is appropriate severity for patients of such young age. After negative results of gene panel sequencing and MLPA, GS was performed and revealed mosaic gross deletion of chr8:118798015_118893064del affecting exons 2–11 in the *EXT1* gene (5/43 = 11.6% of the total read count) (Figures 2A, B). Deletion was confirmed by Sanger sequencing (Figure 2C).

Both families were recommended to regularly monitor the size of the osteochondromas and bone deformities in order to take timely and appropriate orthopedic and surgical measures for correction.

## 4 Discussion

We presented two cases of mosaic partial deletion of the *EXT1* gene. To the best of our knowledge, to date, only six cases with mosaic variants in the *EXT1* and *EXT2* genes are reported in three publications (Szuhai et al., 2010; Sarrión et al., 2013; Oliver et al., 2019). The most frequent (three out of six cases) are mosaic deletions of exons 2–11 of the *EXT1* gene, one mosaic deletion of exons 2–3 in the *EXT1* gene, and deletion of exon 2 of the *EXT2* gene (Szuhai et al., 2010; Sarrión et al., 2013). The last of previously described mosaic variants was a deletion affecting exon 1 of the *EXT1* gene and last exons of the *SAMD12* gene; this deletion led to a fusion between two gene transcripts and was detected by RNA-seq (Oliver et al., 2019).

One of our patients had a recurrent mosaic deletion of exons 2–11 of the *EXT1* gene, while another individual had a novel mosaic deletion of exons 8–11 of the same gene. Both children had disease onset at the age of 3–3.5 years when the first osteochondroma was discovered. One of them had more than 10 osteochondromas at the age of 11 years, with deformation of radial bones that correspond to the average moderate severity of MO at this age. It is interesting to note that despite the low percentage of mosaicism observed in all cases, including ours, the clinical presentation of patients does not differ significantly in severity from non-mosaic cases (Szuhai et al., 2010; Sarrión et al., 2013; Oliver et al., 2019). Both of our probands underwent standard management for MO, which includes periodic evaluation by orthopedic specialists and surgical intervention based on functional limitations or symptomatic presentation caused by osteochondromas. One patient underwent multiple surgical procedures, whereas the younger proband did not require surgical intervention.

Mosaic variants are also intriguing in the context of the two-hit hypothesis of MO pathogenesis (Bovée et al., 1999). According to this hypothesis, probands may harbor an additional somatic loss-of-function variant in the *EXT1* gene, contributing to osteochondroma formation. Furthermore, it is plausible that the percentage of mosaic mutant alleles in osteochondromas could be higher, potentially explaining similarities in disease progression between mosaic and germline variants. Unfortunately, neither we nor other researchers had access to tumor samples to investigate the level of mosaicism of the identified variants or explore potential second somatic variants.

All reported mosaic cases of MO are structural variants, particularly deletions, with no instances of mosaic SNVs reported to date. Among eight reported mosaic CNVs, including our own findings, four CNVs are deletions affecting exons 2–11 of the *EXT1* gene (Szuhai et al., 2010; Sarrión et al., 2013; Oliver et al., 2019). Although germline deletions of exons 2–11 have been noted in various cohort studies, in our cohort study, the deletion of exons 2–11 was counted for 4 cases out of 10 pathogenic CNVs in the *EXT1* gene (Vink et al., 2004; Sarrión et al., 2013; Li et al., 2018; Borovikov et al., 2024). Breakpoint details are available for two germline deletions and two mosaic deletions affecting exons 2–11 of the *EXT1* gene (Figure 2A) (Szuhai et al., 2010; Jennes et al., 2011). The deletion of exons 2–11 of the *EXT1* gene identified in our study (case 2) is the smallest, spanning 95,049 base pairs. Furthermore, each deletion displays distinct breakpoints, suggesting an absence of hotspot regions for breakpoint junctions. Multiple sites for breakpoints due to the size of intron 1 (272,882 bp) of the *EXT1* gene (312,457 bp) could correspond to the higher prevalence of deletion events affecting exons 2–11 among all germline and mosaic CNVs.

In both cases where CNVs were identified, we observed the absence of long homologous sequences or closely located repeats in breakpoint regions of the same class. The deletion spanning exons 8–11 exhibited a 2-base microhomology, while the deletion spanning exons 2–11 displayed a 3-base microhomology. These findings suggest that non-homologous end joining (NHEJ) is the most plausible mechanism for both deletions as NHEJ typically requires short homologous sequences, typically 1–4 base pairs in length. Comparatively, another study investigating CNVs in the *EXT1/2* genes reported that 6 out of 10 CNVs likely originate from NHEJ (Jennes et al., 2011).

In both cases analyzed in our study, we observed a mosaic deletion with an approximate allele frequency of 10%. In both cases, retrospective analysis of the MLPA profile could be regarded as suggestive for a mosaic deletion, but the decrease in signals was too subtle (0.71–0.9 for various probes within affected exons) to detect with the routinely used parameters (dosage quotient for a normal copy number between 0.80 < DQ < 1.20). In case 1, mosaic deletion was initially detected through RNA analysis in fibroblast culture, revealing allelic imbalance (45:55) in the *EXT1* gene. This distribution of alleles corresponded with the count of deletion-supporting reads in the GS data, thereby validating the effect of the deletion at the RNA level. However, the observed difference in allele expression revealed by RNA analysis may not have been adequately discerned solely due to the presence of a mosaic deletion, highlighting the crucial role of GS data in this validation process.

Routine diagnostic methods often struggle to detect such variants, and even RNA analysis may not always be conclusive. Additionally, genome sequencing has limitations, particularly in achieving sufficient coverage for accurately identifying variants with low mosaic frequencies. This underscores the need for caution in interpreting our findings as they raise intriguing questions about the pathogenic threshold of mosaic variants, particularly given their rarity in most hereditary diseases. Future investigations on this subject would greatly benefit from access to more patient samples exhibiting similar characteristics.

In conclusion, our study contributes two novel cases of mosaic partial deletions of the *EXT1* gene, adding to a limited pool of reported mosaic variants in MO. Despite the rarity of mosaic events, the clinical severity of these cases remains comparable to non-mosaic cases. The predominance of deletion events in mosaic variants underscores the distinct genetic landscape of MO, highlighting the need for further investigation into the genetic mechanisms underlying this condition. Future research with larger cohorts and access to tumor samples could provide deeper insights into the prevalence and impact of mosaic events in MO pathogenesis and clinical outcomes.

## Data Availability

The datasets for this article are not publicly available due to concerns regarding participant/patient anonymity. Requests to access the datasets should be directed to the corresponding author.
